# Psychological effects of PTSD and major depression following the wildfires in Fort McMurray: A fifth-year post-disaster study

**DOI:** 10.1192/j.eurpsy.2023.1010

**Published:** 2023-07-19

**Authors:** W. Mao, M. Adu, E. Eboreime, R. Shalaby, N. Nkire, B. Agyapong, H. Pazderka, G. Obuobi-Donkor, E. Owusu, F. Oluwasina, Y. Zhang, V. Agyapong

**Affiliations:** 1 Psychiatry, University of Alberta, Edmonton; 2 Psychiatry, Dalhousie University, Nova Scotia, Canada

## Abstract

**Introduction:**

As a result of the wildfire that engulfed Fort McMurray (FMM), Alberta, Canada in May 2016, over 90,000 residents were evacuated from the city. Approximately 2400 homes, or 10% of the housing stock, were destroyed in Fort McMurray. About 200,000 hectors of forest were destroyed by the fire, which reached into Saskatchewan. In the aftermath of a major disaster, a community’s infrastructure is disrupted, and psychological, economic, and environmental effects can last for many years.

**Objectives:**

Intensive research was conducted in Fort McMurray five years after the wildfire disaster to determine the prevalence of major depressive disorder (MDD) and post-traumatic stress disorder (PTSD) among residents of the community and to determine the demographic, clinical, and other risk factors of probable MDD and PTSD.

**Methods:**

An online questionnaire administered via REDCap was used to collect data in a quantitative cross-sectional study between 24 April and 2 June 2021. Patients were asked to complete the Patient Health Questionnaire (PHQ-9) in order to assess the presence of symptoms associated with MDD. An assessment of likely PTSD in respondents was conducted using the PTSD Checklist for DSM-5 (PCL-C). In this study, descriptive, univariate, and multivariate regression analyses were conducted.

**Results:**

Out of 249 people who accessed the survey link, 186 completed it (74.7% response rate). There was a median age of 42 among the subscribers. A majority of the sample consisted of 159 (85.5%) females; 98 (52.7%) over the age of 40; 136 (71%) in a relationship; and 175 (94.1%) employed. Our study sample had an overall prevalence of 45.0% (76) of MDD symptoms. The multivariate logistic regression model revealed four variables that were independently associated with MDD symptoms, including being unemployed, diagnosed with MDD, taking sedative-hypnotics, and willingness to receive mental health counseling. A total of 39.6% of our respondents (65) reported having likely PTSD. Three independent variables: received a mental health depression diagnosis from a health professional, would like to receive mental health counseling, and have only limited or no support from familycontributed significantly to the model for predicting likely PTSD among respondents while controlling the other factors in the regression model.

**Conclusions:**

The findings of this study indicate that unemployment, the use of sleeping pills, the presence of a previous depression diagnosis, and the willingness to seek mental health counseling are significant factors associated with the increase in the prevalence of MDD and PTSD following wildfires. Support from family members may prevent these conditions from developing.

**Disclosure of Interest:**

None Declared

